# Electronic Versus Paper-Based Assessment of Health-Related Quality of Life Specific to HIV Disease: Reliability Study of the PROQOL-HIV Questionnaire

**DOI:** 10.2196/jmir.3330

**Published:** 2014-04-25

**Authors:** Martin Duracinsky, Christophe Lalanne, Cécile Goujard, Susan Herrmann, Christian Cheung-Lung, Jean-Paul Brosseau, Yannick Schwartz, Olivier Chassany

**Affiliations:** ^1^Université Paris-Diderot, Sorbonne Paris-CitéUnité de Méthodologie des critères d’évaluation (Patient-Reported Outcomes)EA Recherche Clinique Coordonnée Ville-Hôpital, Méthodologies et Société (ED 393)Paris cedex 10France; ^2^Hopital Universitaire de BicetreService de Médecine Interne et de Maladies InfectieusesAssistance Publique - Hopitaux de Paris (AP-HP)Le Kremlin-BicetreFrance; ^3^Inserm Unit UMR-SO 669University Paris Sud, Paris DescartesParisFrance; ^4^Paris DescartesUniversity Paris SudLe Kremlin-BicêtreFrance; ^5^Institute for Immunology and Infectious DiseasesMurdoch University, Western AustraliaPerthAustralia; ^6^Fondation Maison des champsService ACT du Val de MarneVal de MarneFrance; ^7^Parietal TeamInria Saclay–Ile de FranceSaclayFrance; ^8^NeurospinCEA/DSV/I2BMGif-sur-YvetteFrance; ^9^Department of Clinical ResearchSaint-Louis HospitalAP-HPParisFrance

**Keywords:** HIV, quality of life, patient-reported outcomes, electronic records, reliability

## Abstract

**Background:**

Electronic patient-reported outcomes (PRO) provide quick and usually reliable assessments of patients’ health-related quality of life (HRQL).

**Objective:**

An electronic version of the Patient-Reported Outcomes Quality of Life-human immunodeficiency virus (PROQOL-HIV) questionnaire was developed, and its face validity and reliability were assessed using standard psychometric methods.

**Methods:**

A sample of 80 French outpatients (66% male, 52/79; mean age 46.7 years, SD 10.9) were recruited. Paper-based and electronic questionnaires were completed in a randomized crossover design (2-7 day interval). Biomedical data were collected. Questionnaire version and order effects were tested on full-scale scores in a 2-way ANOVA with patients as random effects. Test-retest reliability was evaluated using Pearson and intraclass correlation coefficients (ICC, with 95% confidence interval) for each dimension. Usability testing was carried out from patients’ survey reports, specifically, general satisfaction, ease of completion, quality and clarity of user interface, and motivation to participate in follow-up PROQOL-HIV electronic assessments.

**Results:**

Questionnaire version and administration order effects (N=59 complete cases) were not significant at the 5% level, and no interaction was found between these 2 factors (*P*=.94). Reliability indexes were acceptable, with Pearson correlations greater than .7 and ICCs ranging from .708 to .939; scores were not statistically different between the two versions. A total of 63 (79%) complete patients’ survey reports were available, and 55% of patients (30/55) reported being satisfied and interested in electronic assessment of their HRQL in clinical follow-up. Individual ratings of PROQOL-HIV user interface (85%-100% of positive responses) confirmed user interface clarity and usability.

**Conclusions:**

The electronic PROQOL-HIV introduces minor modifications to the original paper-based version, following International Society for Pharmacoeconomics and Outcomes Research (ISPOR) ePRO Task Force guidelines, and shows good reliability and face validity. Patients can complete the computerized PROQOL-HIV questionnaire and the scores from the paper or electronic versions share comparable accuracy and interpretation.

## Introduction

Patient reports are useful to “recall observations, to inform others, to instruct students, to gain knowledge, to monitor performance, and to justify interventions” [1] and form part of clinical decision-making. With the advent of modern technologies, electronic health records (eg, biological measures, treatments, imaging results) are used increasingly, especially in managing chronic disease [2]. Information such as pain, fatigue, depression, and health-related quality of life (HRQL) are also gathered through self-reported measures, and are known as patient-reported outcomes (PRO) [3]. Gathering this information electronically is becoming increasingly common because electronic assessment provides users with direct feedback including secure storage in databases supporting access controls and role-based permissions, lower administrative costs, and easier follow-up of patients’ records over time. Moreover, electronic diaries or electronic PRO (ePRO) measurement provide quick, convenient, and reliable assessment of patients’ HRQL, and improve compliance with self-assessed HRQL [4] or at least help to understand reasons for noncompliance [5]. Because responses can be enforced (eg, patients are not able to submit a Web form or possibly skip to the next questionnaire if some of the questions were not answered), ePROs help to reduce the problem of missing data [6].

There is compelling evidence that electronic and paper-and-pencil PROs deliver equivalent measures [7], and sometimes electronic ones are more reliable [8,9], although some discrepancies between paper and electronic versions of the same questionnaire have been reported [10]. However, the Internet is used increasingly to seek information related to symptoms, HRQL, drug adverse events, or simply share self-experience with chronic disease. Human immunodeficiency virus (HIV) disease is now considered a chronic disease with costly treatment, and researchers are currently seeking solutions to optimize HRQL assessments not only in clinical trials, but also in clinical routine care. For example, intervention studies have demonstrated that adherence could be improved when monitored via mobile phones [11,12], suggesting that electronic health records and care management systems are promising approaches to improving HIV care.

Demonstrating equivalence between electronic and paper versions of PRO measurement is an essential step when a validated paper instrument is migrated to an electronic format, especially when both versions are to be used interchangeably. According to recent International Society for Pharmacoeconomics and Outcomes Research (ISPOR) guidelines [13], an ePRO questionnaire should deliver comparable or better data compared to a paper-and-pencil questionnaire, and the measurement of difference between the 2 data gathering methods is an essential feature of validation.

An electronic version of the Patient-Reported Outcomes Quality of Life-HIV (PROQOL-HIV) questionnaire [14,15] was developed to meet the challenges of the electronic health measures era. The aim of this study was to study the psychometric properties, especially face validity and reliability, of the electronic version of the PROQOL-HIV questionnaire and to suggest further refinements to the Web interface based on participant input and the technical issues encountered during the validation study. This study provides details about the participants, study setup, and principal results for HRQL data as well as users’ feedback.

## Methods

### Recruitment

The study was conducted in 2 centers in France: the Kremlin Bicêtre hospital (Assistance Publique-Hôpitaux de Paris) and the institutional apartments for people living with HIV, *Service ACT du Val de Marne, Fondation Maison des champs*.

Informed consent was obtained from all participants. The study was approved by a local ethics committee. Storage of individual patient data on a dedicated server was also approved by an independent French administrative authority in charge of personal data registration and protection (CNIL record #1566050). Inclusion criteria were French-speaking, HIV-seropositive outpatients receiving routine HIV clinical care, aged 18 years or older. People were excluded from the study if they attended the hospital for urgent care or were hospitalized with HIV-related illness.

### Measurement Instrument

The PROQOL-HIV questionnaire [14,15] is composed of 43 Likert-type items (5-point scale ranging from 0=never to 4=always), including 39 items targeting 8 domains of HRQL and general health: physical health and symptoms (9 items), treatment impact (10 items), emotional distress (4 items), health concerns (4 items), body change (4 items), intimate relationships (3 items), social relationships (2 items), and stigma (2 items). Four extra items dealing with religious beliefs, finance, having children, and satisfaction with care are not part of the scoring scheme, but are used to gather additional information from the respondent. This questionnaire has been shown to exhibit good reliability with Cronbach alpha ranging from .772 to .885 and intraclass correlations greater than .7 for all dimensions with more than 2 items. Responses to items were totaled for each dimension and standardized on a scale from 0 to 100 points, in which higher values indicate a better health state.

The psychometric validation of the PROQOL-HIV questionnaire [15] included 123 French participants. In this sample, the estimated standard deviation for the physical health and symptoms scale was 20.1 points, with a Cronbach alpha of .885, suggesting that the standard error of measurement approached 6.8 points. This shows individual scores with a half-width confidence interval (or margin of error) of 13 points.

### Web Interface

**Figure 1:**
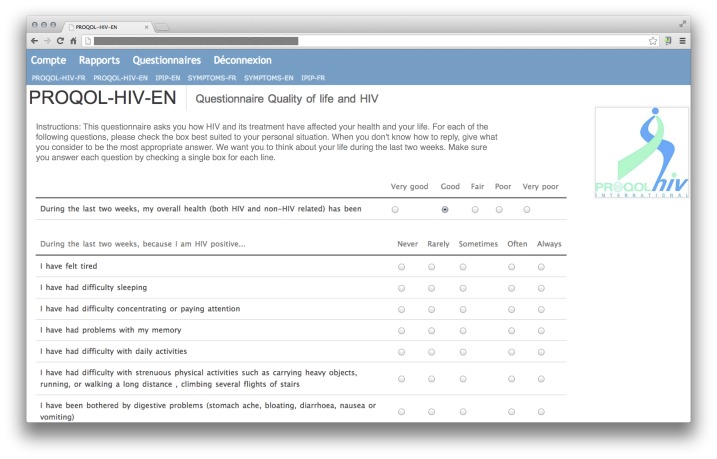
Screenshot of the Web interface for the English version of the PROQOL-HIV questionnaire.

The Web interface was developed using the Python programming language and data were stored in a PostgreSQL database. The system makes use of dynamic Hypertext Markup Language (HTML; JQuery) to save answers instantly or highlight missing responses when validating entries. The Web version of the PROQOL-HIV questionnaire was developed to replicate the paper-based questionnaire as closely as possible. Compared with the original paper-based version, only minor modifications were made: the entire questionnaire was presented on a single HTML page (rather than 2 separate sheets of paper), and response options were presented as radio buttons on a horizontal grid (instead of checkboxes) with headings aligned on top of each section of the questionnaire. A sample screenshot of the Web questionnaire is provided (Figure 1). Before completing the Web form, users registered with a personal username and password on a log-in page. If no activity was detected after 5 minutes, the session was timed out to ensure data was protected. The uncompleted questionnaires were saved, but were not used in the analysis. Responses, timestamp, and username were saved in a secure database. Individual timestamps were kept for each selected response options, not simply for the questionnaire as a whole. Scale scores were computed directly on the server and were visually presented to the user at the end of the session through bar charts and a numerical table. For the purpose of this validation study, anonymized individual data were extracted from the database.

### Administration

Each patient received an information sheet describing the aim of the study, detailed instructions on how to connect to the website with a secure password, and a brief questionnaire to fill in about basic demographic and clinical information. Biological variables, such as viral load or lymphocyte CD4 count, were retrieved from patients’ files. The paper and electronic HRQL questionnaires were completed in a randomized crossover design, with a 2- to 7-day interval. At the first meeting, patients completed the clinical and demographic questionnaire with the help of the nursing staff if needed. They were then asked to complete either the PROQOL-HIV paper version or the electronic version on a dedicated computer at the hospital, depending on the sequence to which they were randomized. A randomization list was established using a computer program (shuffled balanced binomial draws, as in 2-arm treatment allocation) before the beginning of the study. Each new patient was allocated to the next sequence in the randomization list. Patients unable to complete the electronic questionnaire at home (eg, lack of Internet access) were switched from the electronic to the paper group. All patients were then recalled to complete the second questionnaire at home from the next day up to 1 week later. Satisfaction with the electronic questionnaire and general impressions regarding the user interface were assessed directly after completing the Web version of the PROQOL-HIV questionnaire. Each patient also provided their general impression and preference through a separate survey on a paper sheet at the end of the study period, and was asked to report any clinical events in the intervening period. This was returned by the prepaid envelope. They were also asked to rate the clarity of presentation, readability of questions and response options, and ease of use of the website. Participants randomized to the paper-based HRQL assessment in the second round mailed their completed questionnaires to the researcher. Cognitive debriefing was carried out on 10 patients who were administered the electronic version at the hospital.

### Statistical Analysis

#### Statistical Tests

Biomedical and demographic data were summarized using classical descriptive statistical indicators of central location and dispersion. Group comparisons were performed using Wilcoxon-Mann-Whitney and Pearson chi-square tests for numerical and categorical variables, respectively, to assess heterogeneity between centers. Comparisons of scale scores for the paper and electronic versions were performed using nonparametric Wilcoxon test for paired samples. The effects of form (electronic vs paper) and order of presentation or sequence (electronic first or paper first) were tested on physical health and symptoms scores (9 items with total score expressed on a scale of 0-100 points) in a 2-way ANOVA with patients as random effects. The physical health and symptoms dimension was chosen because it has a high number of items and was shown to explain more of the total variance in factor analysis when validating this questionnaire [15]. However, similar analyses were also carried out on full-scale scores (39 items, 0-100 points). Alternate form and test-retest reliability were evaluated using Pearson and intraclass correlations for each dimension. A bootstrap procedure (B=500 replicates) was used to compute the 95% confidence interval for the ICC. This last measure of consistency, or temporal stability, was considered to determine the number of participants in a preliminary power analysis.

Usability testing used data from patients’ survey reports (ease of completion, quality, and clarity of user interface, and motivation for on-going monitoring of HRQL). In addition, analysis of response time per item was carried out based on available individual timestamps. All statistical analyses were done using the R statistical software, version 2.15 (The R Project for Statistical Computing).

#### Power

From the validation study of the PROQOL-HIV questionnaire, which included a test-retest analysis on 34 French patients (average interval=52 days), the intraclass correlation was estimated at .859 (95% CI .710-.960) for full-scale scores [15]. Considering a theoretical reliability of 0.8, a sample size of 65 individuals is sufficient to verify if the ICC is greater than .75 with a 95% confidence interval [16].

## Results

### Participants

**Figure 2:**
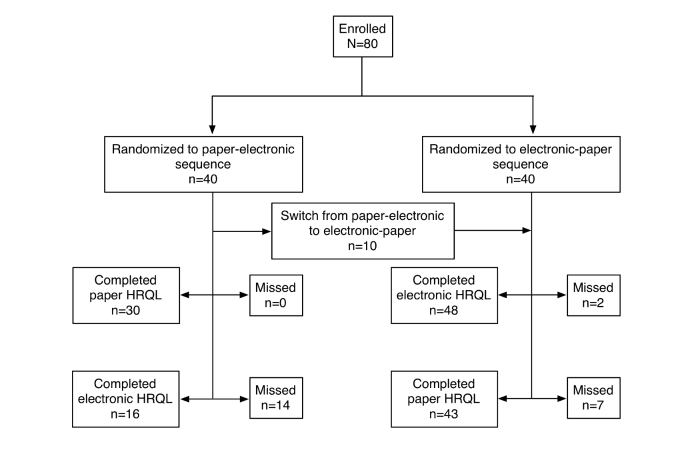
Participant flowchart.

A sample of 80 outpatients (male: 52/79, 66%; female: 27/79, 34%) with a mean age of 47 years (SD 10.9) were enrolled in this study. Most participants were enrolled from the hospital (70/79, 88%). A flowchart demonstrating the randomization procedure is provided (Figure 2). Patients were randomized to 1 of 2 groups (paper version first or electronic version first) when they entered the study. However, because some patients reported having no Internet connection available at home, there were 10 switches in the order of administration (patients allocated to the group paper version first were given the electronic version first).

Overall, two-thirds of the participants were native French speakers (51/80, 64%). The main characteristics of the patients were stratified by center and summarized (Table 1). Participants from the institutional apartments were more likely to be living alone and to be without a professional activity. Their HIV-related immune decline was more advanced (56%, 5/9) stage C, average CD4 counts <500 cells/mm^3^) compared with patients enrolled at the hospital. Overall, 89% (70/79) of patients were treated with an antiretroviral treatment (ART). Regarding viral hepatitis co-infection, 18% (14/79) of patients were seropositive for hepatitis C and 6% (5/79) for hepatitis B. Antidepressants were the most common concomitant treatment (14%,11/79 overall). None of the participants reported clinical events in the interval between the 2 study time points.

**Table 1:** Demographic and biomedical information on study participants.

Variable		N	Hospital (n=70)	Institution (n=10)	All patients	*P* ^b^
**Age (years)**		79				.58
	Mean (SD)		46.9 (11.2)	44.4 (8.9)	46.7 (10.9)	
	IQR		39.5-53.0	37.0-51.0	39.0-53.0	
**Gender, n (%)**		79				.12
	Male		44 (63)	8 (89)	52 (66)	
	Female		26 (37)	1 (11)	27 (34)	
Not currently working		78	15 (21)	6 (75)	21 (27)	.005
Education level (university), n (%)		78	29 (41)	4 (50)	33 (42)	.93
Marital status (single), n (%)		77	33 (48)	5 (62)	38 (49)	.68
Living alone, n (%)		78	31 (44)	8 (100)	39 (50)	.009
**Comorbidities, n (%)**						
	Depression	78	9 (13)	3 (38)	12 (15)	.07
	Psychiatric disorder	78	1 (1)	0 (0)	1 (1)	.73
	Cardiovascular disease	78	9 (13)	3 (38)	12 (15)	.07
	Diabetes	78	6 (9)	1 (12)	7 (9)	.71
	Other comorbidities	78	5 (7)	0 (0)	5 (6)	.43
	Lipodystrophy	79	15 (21)	2 (22)	17 (22)	.96
**Current treatment, n (%)**		78				
	Prophylaxis^c^		3 (4)	3 (38)	6 (8)	<.001
	Antidepressant		8 (11)	3 (38)	11 (14)	.045
	Lipid-lowering		7 (10)	0 (0)	7 (9)	.35
**Year of diagnosis**		78				.19
	Mean (SD)		1998 (8)	2002 (7)	1999 (8)	
	IQR		1990-2005	2000-2008	1991-2006	
HAART status, n (%)		79	61 (87)	9 (90)	70 (89)	.99
**CDC stage, n (%)**		79				
	A (asymptomatic)		41 (59)	2 (22)	43 (54)	.07
	B (symptomatic conditions)		13 (19)	2 (22)	15 (19)	
	C (AIDS-indicator conditions)		16 (23)	5 (56)	21 (27)	
**Year of first HAART**		66				.02
	Mean (SD)		2002 (6)	2008 (3)	2003 (6)	
	IQR		1996-2007	2007-2009	1997-2007	
**Viral co-infection, n (%)**						
	Hepatitis C	79	14 (20)	0 (0)	14 (18)	.31
	Hepatitis B	79	4 (6)	1 (10)	5 (6)	.99
**CD4**						
	CD4 counts (cells/mm^3^), mean (SD)	79	623 (438)	407 (191)	598 (422)	.04
	IQR		441-700	213-527	424-694	
	CD4 %, mean (SD)	74	30.3 (10.2)	20.2 (9.8)	29.6 (10.4)	—
	IQR		24-37	19-24	23-37	
Viral load (undetectable)		21	1 (8)	8 (89)	9 (43)	.001

^a^HAART: highly active antiretroviral therapy; CD4: cluster of differentiation 4, T helper cells playing a major role in the human immune system; CDC: Centers for Disease Control and Prevention (classification system for HIV-infected adults and adolescents).

^b^Wilcoxon-Mann-Whitney test for 2 independent samples and Pearson chi-square.

^c^Toxoplasmosis, pneumocystis.

### Health-Related Quality of Life Data

A total of 64 of 80 electronic surveys were available at the end of the study. Of these, 50 participants completed all 43 items. In all, 9 participants (11%) were naive to ART; therefore, they were not required to complete the 10 treatment-related items. Three patients filled in only part of the questionnaire and were excluded from further analyses. Regarding the paper version, there were 73 records, including 49 full records, 10 ART-naive patients, and 14 partially filled records. A total of 59 electronic and paper questionnaires were available for test-retest analysis (Figure 2).There was a greater number of missing questionnaires for the paper version (n=14) compared to the electronic version (n=3, 2-sample test for equality of proportions with continuity correction, *P*=.02). This might be explained by the fact that people forgot to fill the questionnaire at home or there were problems with mailing the questionnaires. Regarding missing responses in the paper-based questionnaires, there were no more than 5% (2/43) of missing items for very few cases (n=3), and they were imputed by individual mean scores for each dimension.

**Figure 3:**
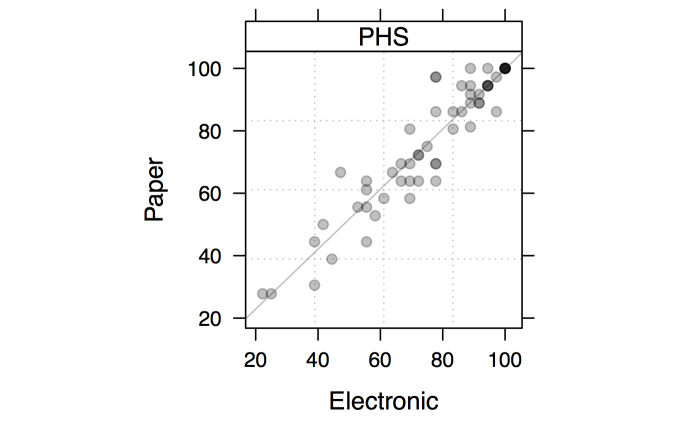
Individual scores (N=59) on electronic and paper versions of the PROQOL-HIV questionnaire for the physical health and symptoms (PHS) dimension. Individual points are displayed with alpha transparency so that darker symbols indicate a higher number of identical pairs of scores. The straight line represents the ordinary least squares regression line.

The distribution of individual standardized scale scores on the physical health and symptoms dimension (0-100 points, where higher values reflect a better HRQL state) are illustrated as a 2-way scatter display (Figure 3). Summary statistics for all paper and electronic scale scores were computed for each dimension (Table 2). The lower level of agreement for the general health scale (Spearman ρ=.72, ICC=.714) may be because there are only 5 possible scores for this single item. In the present study, standard deviations for the physical health and symptoms scale (paper: SD 20.8; electronic: SD 20.3) were close to what was observed previously.

**Table 2:** Reliability analysis.

Domain (n of items)	Paper		Electronic		*r* (ρ)	ICC (95% CI)	*P* ^a^
	Mean (SD)	IQR	Mean (SD)	IQR			
All items^b^ (38)	68.3 (16.5)	54.1-82.1	69.5 (16.2)	56.7-84.4	.868 (.851)	.868 (.815-.931)	.21
Body concerns (4)	78.0 (26.1)	54.7-100.0	76.9 (26.0)	56.2-100.0	.827 (.777)	.827 (.708-.955)	.30
Emotional distress (4)	68.8 (26.9)	50.0-93.8	69.4 (26.0)	50.0-93.8	.842 (.874)	.842 (.772-.929)	.97
General health (1)	23.5 (20.7)	0.0-25.0	21.4 (21.7)	0.0-25.0	.715 (.790)	.714 (.550-.881)	.46
Health concerns (4)	51.6 (28.3)	25.0-81.2	55.6 (29.4)	31.2-81.2	.785 (.767)	.785 (.680-.884)	.46
Intimate relationships (3)	57.4 (33.8)	33.3-85.4	63.4 (31.1)	39.6-91.7	.782 (.782)	.779 (.625-.909)	.05
Physical health and symptoms (9)	76.3 (20.8)	63.9-94.4	75.6 (20.3)	63.9-91.7	.940 (.923)	.939 (.905-.979)	.63
Social relationships (2)	80.8 (27.1)	75.0-100.0	80.4 (28.9)	62.5-100.0	.824 (.803)	.822 (.725-.941)	.99
Stigma (2)	33.3 (33.8)	0.0-50.0	36.4 (34.5)	0.0-50.0	.712 (.741)	.712 (.543-.893)	.25
Treatment impact (10)	71.7 (21.2)	57.5-90.0	72.2 (20.9)	61.2-90.0	.708 (.783)	.708 (.444-.982)	.94

^a^Using Wilcoxon signed rank test.

^b^Full-scale score was calculated following the exclusion of 4 extra items and the general health item.

A random-effects 2-way ANOVA was used to assess the effects of the type of questionnaire (electronic or paper) and the order of administration. A total of 59 complete cases (74% of participants) were available for this analysis (Figure 2). It is worth noting that the order of administration was not balanced because 28% of participants ended up filling out the paper version first then the electronic version. No interaction between the type of questionnaire and administration order was found (*F*_1,55_=0.098, *P*=.76). Likewise, there was no effect of the type of questionnaire (*F*_1,55_=0.529, *P*=.47) or administration order (*F*_1,76_=0.942, *P*=.34). These results indicate that scores obtained on either the electronic- or paper-based version were not statistically different, independent of the order of administration. Analysis based on full-scale scores yielded similar results (data not shown).

**Figure 4:**
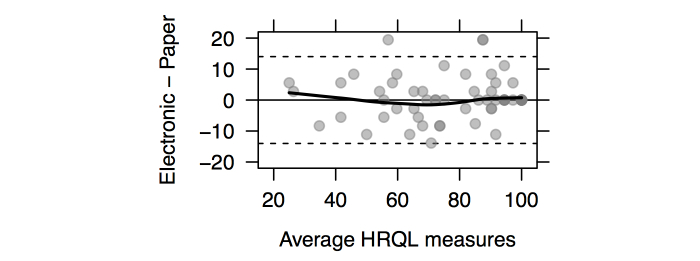
Bland-Altman plot showing the difference between scores of the electronic and paper versions as a function of average physical health and symptoms scores. The upper and lower dashed horizontal lines represent limits of agreement. A Lowess smoother (solid thicker black line) has been added to differentiate local irregularities on the distribution of scores.

Reliability indexes (Table 2) were in the acceptable range, with Pearson correlations greater than .7 and intraclass correlation ranging from .708 (treatment impact) to .939 (physical state and symptoms). Mean scores for each dimension were not significantly different according to Wilcoxon signed rank tests for paired samples, even without considering correction for multiple testing (Bonferroni method). This suggests that, on average, this sample demonstrates comparable HRQL on all dimensions. The joint distribution of individual scores obtained from electronic and paper versions of the questionnaire for the physical and health symptoms dimension was analyzed separately (Figure 3). It is worth noting that although mean scores were slightly different between the paper (76.3 points) and electronic (75.6 points) versions, this difference was not significant (*P*=.63; Table 2) and would not be considered as clinically relevant anyway. A Bland-Altman chart is provided in Figure 4 with limits of agreement computed as ±1.96×SD, where SD is the standard deviation for the difference between individual scores of the two versions. In both cases, it can be seen that scores are generally close to one another, with the exception of 3 participants who had higher physical health and symptoms scores on the electronic version compared to the paper version.

### Usability Analysis

Results on the usability and satisfaction survey were analyzed separately (Table 3). Of the 79% (63/80) of surveys that were completed, two-thirds of participants (65%, 36/60) reported that they would be interested in electronic assessment of their HRQL by using an electronic version of PROQOL-HIV in routine clinical care. Overall, 55% of the participants indicated a preference toward the electronic questionnaire compared to the paper-based questionnaire. Only 9% (5/55) of participants preferred the paper version, and 36% (20/55) were indifferent. Regarding the version preferred by patients, there were no significant associations with age (*P*=.12), gender (*P*=.60), marital status (*P*=.39), living mode (alone, *P*=.48), education level (*P*=.18), professional activity (*P*=.59), and diagnosis date (*P*=.39). Individual ratings of the PROQOL-HIV user interface (80% to 100% of positive responses) confirmed the clarity and usability of the Web version, although the visual display of summary scores might need further improvement as confirmed by 10 cognitive debriefings that were carried out with patients.

**Table 3:** Participants’ survey responses.

Question		N	n (%)	Mean (SD)	IQR
Display on screen is comprehensible and easy to follow		63	63 (100)		
Font size looks appropriate		62	61 (98)		
Single page design is satisfactory		62	62 (100)		
Visualization of results is an interesting option		61	58 (95)		
Display of results is comprehensible		60	48 (80)		
Ease of input responses		63		4.6 (0.7)	4-5
Questions readability		63		4.6 (0.8)	4-5
Scores readability		61		4.0 (1.0)	4-5
Interested in longitudinal follow-up of personal scores		60		4.4 (0.9)	4-5
Difficulty with computing material		60	8 (13)		
Ease of filling (electronic vs paper)		55		4.0 (1.0)	3-5
**Preference**		55			
	Indifferent		20 (36)		
	Paper		5 (9)		
	Electronic		30 (55)		

### Analysis of Response Time

The median time to complete the electronic version of PROQOL-HIV was 7 minutes, with 50% of the times between 4.8 and 11.1 minutes. In 2 cases, participants took more than 20 minutes to complete the electronic questionnaire; in 16 cases, participants took less than 5 minutes. The former was explained by disconnection from the hospital network. In the latter case, respondents had completed the paper questionnaire previously and were familiar with the items. Mean individual response time (trimmed to 5%) per item was 9.1 seconds on average (range 3.7-20.1). Only 17 of 54 (31%) participants completed the questionnaire in the order presented. For those participants who provided answers in a different order, it should be noted that filling order was usually altered for one question, but not for more than 6 questions in total. This included participants who delayed completing the questionnaire for a short period of time or those who forgot to answer some of the questions before submitting the Web form. In the latter case, missing answers were highlighted by the system and participants had to complete them again.

## Discussion

### Principal Results

According to international guidelines, the validation of an electronic version of an existing questionnaire requires the demonstration of equivalent measurement properties between the original paper-based and the electronic versions of the questionnaire. This is achieved through statistical measures of correlation and mean differences between the 2 series of individual scores which can be summarized by the intraclass correlation. However, several threats to ensuring equivalent measures have been reported. In particular, substantial changes to the presentation of items or questions to accommodate screen limitations and poor experience with computer use can alter validity or reliability of scores [7].

Typically, cognitive debriefing is carried out with a small number of participants to verify that the content of the electronic questionnaire is perceived in the same way as that of the paper-based version; test-retest studies are restricted to cases in which significant changes were introduced in the electronic version (eg, questionnaire layout, response options). This study goes beyond standard recommendations (cognitive debriefing) and it provides a quantitative assessment of both test-retest reliability and users’ self-perception of the Web version of the PROQOL-HIV questionnaire.

The demographics of the participants in this study are representative of the population of people living with HIV in this French metropolitan area. In 2011, this population was composed of 67% men and 33% women, and 24% of the patients were non-European Union residents. Among them, 17% originated from sub-Saharan Africa. In the Vespa 2 study, 41% of the participants were not currently working and only 37% considered their financial situation as good [17]. When introducing new technologies to health care, it is important that all populations, including immigrants and people with low income, can benefit from such programs. A heterogeneous sample of patients participated in this study, providing they could read French and they could use the electronic questionnaire even if they did not have Internet at home, because it was available at the hospital directly.

The present findings suggest that assessing health-related quality of life specific of HIV disease on a Web-based platform is easy and reliable, and that the electronic version of the PROQOL-HIV questionnaire fulfills the criteria for migrating a paper-based questionnaire to a computer-based mode of administration. Following their analysis of 46 single studies (of 65 eligible case reports) relying mainly on computer-based assessment (n=31, 67%), Gwaltney and collaborators [7] reported an average correlation between paper-based and electronic assessment of .90 (95% CI .87-.92, n=32) without significant differences from studies relying on intraclass correlation or weighted kappa. Our results suggest that the PROQOL-HIV questionnaire can achieve good test-retest reliability, as measured by an intraclass correlation greater than .8 for the principal dimension of the questionnaire or the full-scale score. Moreover, it demonstrated good face validity according to respondents’ self-perception collected at the end of the study, with more than 80% with a positive opinion toward PROQOL-HIV usability and clarity when assessing HRQL specific to HIV disease. Interestingly, only 9% of the patients indicated that they preferred the paper version at the end of the study. However, this might be a biased indicator because the objective of the study was to validate the electronic version. One of the important findings was the high interest in longitudinal follow-up of personal HRQL scores, suggesting that electronic assessment may be of value in routine clinical care for HIV. The electronic version of PROQOL-HIV has been in use by some French patient associations with positive feedback from the local coordinators. Further studies will determine the value of electronic longitudinal follow-up of self-reported HRQL.

The use of electronic PRO measures in HIV care can offer important implications. First, physicians could benefit from a direct and contemporaneous assessment of a patient’s HRQL at the time of the consultation, which will enhance clinical observation and decisions around treatments. Second, there is a growing interest in patient-centered care, which has been shown to improve perceived health outcomes because patients feel engaged in their health care management. Consequently, the opportunity for patients to record their HRQL at home and to have access to their results, together with their physicians, should enhance the relationship between care providers and the patients.

### Limitations

The analysis of open-ended satisfaction questions highlighted critical issues with using PROQOL-HIV as an electronic HRQL questionnaire. Because the questionnaire was given on a single HTML page, response headings for some of the items were not always visible depending on screen size and length of that section. Occasional breakdown of the Internet connection was reported in one center; hence, patients’ responses were not taken into account and they had to fill in the questionnaire again. Display of HRQL summary scores should be complemented by a brief overview of the patients’ health state. Another limitation of the study was the imbalance in the order of administration, although this did not affect the validity of the present findings because there was enough data to analyze the temporal stability of HRQL scores and to compute reliable indicators of participants’ impressions regarding the Web version of PROQOL-HIV. Finally, no systematic pattern was detected for patients who did not follow the order of the questions, suggesting that this does not affect the structure of the PROQOL-HIV questionnaire. It should be noted that patients could also fill in the PRO paper-based questionnaire in random order, but this could obviously not be detected. The absence of follow-up data to study the responsiveness of the electronic PROQOL-HIV and to collect information on how to best display summary scores for easy monitoring of personal data will be assessed in a forthcoming study.

### Conclusions

The PROQOL-HIV instrument has been adapted in a way that would be classified as minor according to ISPOR ePRO Task Force guidelines [12]. The new electronic version shows good reliability and face validity, and scores obtained from paper or electronic modes share comparable accuracy and interpretation. An interesting finding was that few patients (9%), including patients having no Internet at home, preferred the paper-based version of PROQOL-HIV. The desire of participants to have access to the scores of this instrument as a way of tracking themselves over time also shows their interest in Web-based assessments in clinical health care.

## Abbreviations

ART: antiretroviral therapy
HAART: highly active antiretroviral therapy
HIV: human immunodeficiency virus
HRQL: health-related quality of life
HTML: Hypertext Markup Language
ICC: intraclass correlation coefficient
ISPOR: International Society for Pharmacoeconomics and Outcomes Research
PRO: patient-reported outcomes
PROQOL-HIV: Patient-Reported Outcomes Quality of Life-HIV questionnaire
